# Sporogenesis, gametophyte development and embryogenesis in *Glehnia littoralis*

**DOI:** 10.1186/s12870-023-04105-1

**Published:** 2023-02-23

**Authors:** Chunxia Zhou, Kang An, Xin Zhang, Boqiang Tong, Dan Liu, Dongrui Kong, Fuhua Bian

**Affiliations:** 1 College of Life Science, Yantai University, Yantai, 264005 China; 2 Shandong Forestry and Grass Germplasm Resource Center, Jinan, 250102 China; 3 College of Life Science, Ludong University, Yantai, 264025 China

**Keywords:** *Glehnia littoralis*, Sporogenesis, Gametophyte development, Embryogenesis, Abortion

## Abstract

**Background:**

*Glehnia littoralis* is an economic herb with both medicinal and edible uses. It also has important ecological value and special phylogenetic status as it is a monotypic genus species distributing around beach. Little information on its reproductive biology has been reported so far, which has hindered conservation and application of this species. In this study, we observed morphological changes from buds emergence to seeds formation and internal changes during sporogenesis, gametophyte development and embryo and endosperm development of *G. littoralis* using paraffin-embedded-sectioning and stereo microscope.

**Results:**

The results showed that the stages of internal development events of *G. littoralis* corresponded to obvious external morphological changes, most of developmental features were consistent with other Apiaceae species. The development of male and female gametophytes was not synchronized in the same flower, however, exhibited temporal overlap. From mid-late April to mid-May, the anther primordial and ovule primordial developed into the trinucleate pollen grain and eight-nuclear embryo sac, respectively. From late-May to mid-July, the zygote developed into mature embryo. In addition, some defects in gynoecium or ovule development and abnormal embryo and endosperm development were found. We induced that the possible causes of abortion in *G. littoralis* were as follows: nutrient limitation, poor pollination and fertilization, and bad weather.

**Conclusions:**

This study revealed the whole process and morphological characteristics of the development of reproductive organ in *G. littoralis*, which not only provided important data for the study of systematic and conservation biology, but also provided a theoretical basis for cross breeding.

## Background

*Glehnia littoralis* Fr. Schmidt ex Miq., distributed in sandy coastal areas around the North Pacific Ocean, especially the east coast of China, is an endangered perennial herb in Apiaceae. The dried roots of *G. littoralis*, generally called “Bei-Sha-Shen” in China, has high medicinal value for it is usually used in the treatment of several diseases as an important traditional Chinese medicine [1]. In addition, its young leaves and fresh roots are utilized as a vegetable [2, 3]. It has been reported that coumarins, polyacetylenes, lignans, polysaccharides and flavonoids have been isolated from the extracts of *G. littoralis*, which have immunoregulation, anti-inflammatory，antioxidant, antibacterial, antitumor and anticancer activities [2, 4–6]. And the leaf extracts of *G. littoralis* exhibit whitening and anti-wrinkle properties [7].

Wild resources of *G. littoralis* have reduced largely as a result of indiscriminate digging and destruction of beach habitats [8, 9]. It has been listed as a national classII key protected wild plants in China [10]. As medicine and food, *G. littoralis* has been introduced and domesticated for more than 500 years in China, and the genuine area is in Shandong province. However, the quality and yield of *G. littoralis* are major issues which affect the medicinal qualities and economic development. In order to ensure the yield and quality of medicinal materials, it is very necessary to breed new varieties. The comprehensive understanding of plant reproductive biological characteristics can assist in provide premise reference for breeding process and improve the efficiency and quality of breeding. Previous research on *G. littoralis* mainly focused on its active constituents and pharmacological activities [11–13]. In addition, there also have some studies on cultivation technology，genetic diversity and quality control [3, 8, 14–16]. Until now, no systematic report on reproductive biology of *G. littoralis* has been published, which is critical for reveal the characteristics of growth, development and reproduction of plant.

In *G. littoralis*, it is often the case that some of the peripheral florets of the compound inflorescence of dwarf plants become stunted and eventually died. And in the process of fruit development, seed atrophy, embryo or endosperm abnormality and some other phenomena were found in one of mericarp. Therefore, knowledge of embryology and the development of reproductive organs are essential to illuminate these phenomena [17]. Moreover, embryology can provide the basis for understanding reproduction, evolution and endangered mechanism of the plant.

Since little was known about the reproductive biology of *G. littoralis* until now, it is necessary to fully explore its reproductive system, reproductive growth and development in order to better develop the breeding work of this species and explain the internal causes of its endangerment. Therefore, the objectives of this research were: (1) to investigate the development of male and female gametophytes in *G. littoralis*; (2) to understand the process of fertilization and development into mature seeds; and (3) to summarize the correspondence between external developmental features and internal developmental events and the developmental timeline. Ultimately, provide embryological information for the study of reproductive biology of *G. littoralis*.

## Results

### Development of the Anther Wall

**Fig. 1:**
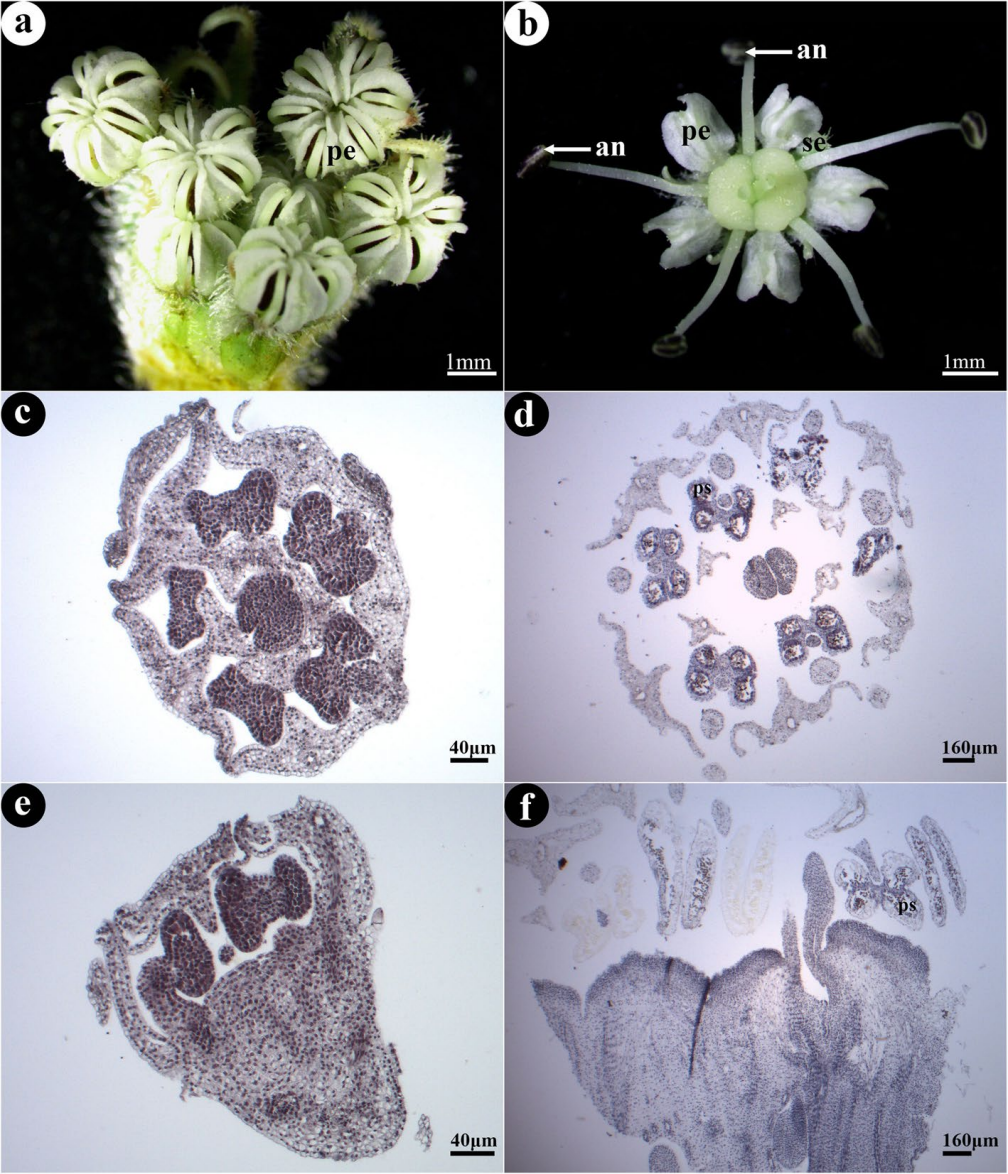
The morphological structure of the flower of *G. littoralis* (show stamens). **a** Flower buds. **b** Blooming flower. **c**, **d** Transverse section of flower bud. **e**, **f** Longitudinal section of flower bud. Se: sepal, pe: petal, an: anther, ps: pollen sac

The flower of *G. littoralis* has five petals, arranged alternately with five separated stamens (Fig. 1a, b, c and f). A few flowers with six petals and six stamens were also found, but not frequently (Fig. 1a and d). Anthers have four distinguishable pollen sacs (Fig. 1d). Due to the central part of petals of *G. littoralis* was wide, they were close to each other and even overlapped in the early, and the anterior part was curved inward and closely together, so that the stamen primordiums curved under the surrounding petals after they grew from the armpit of the sepal (Fig. 1a and e). Then, they stretched out gradually with the development of flowers (Fig. 1b).

**Fig. 2:**
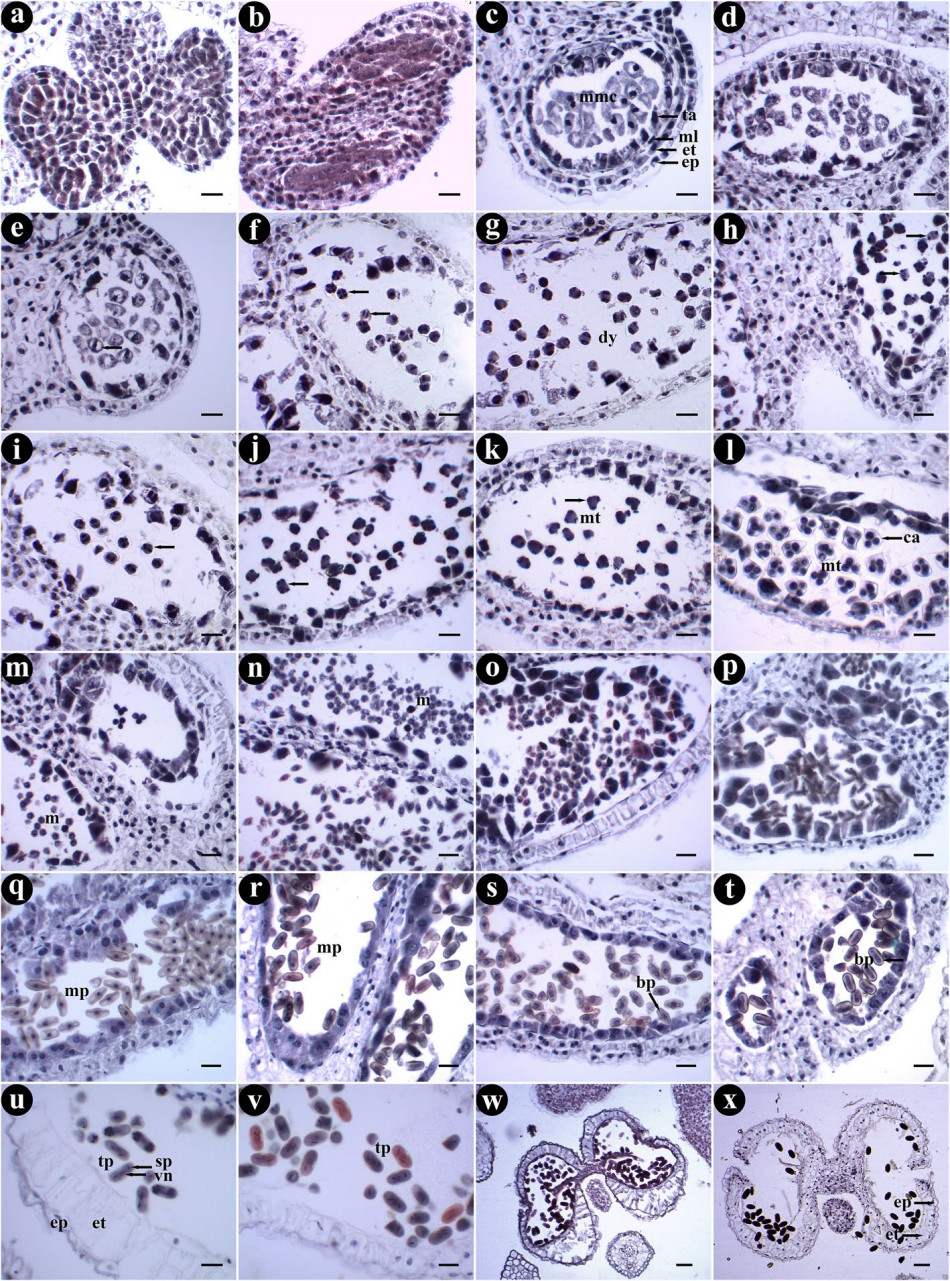
Anther development of *G. littoralis*. **a** Differentiation of anther primordial. **b** Differentiation of archesporial cells. **c** The complete anther wall. **d**-**j** Meiosis of microspore mother cells. **k**, **l** The tetrad of microspores. **m**, **n** microspores released from tetrad. **o**, **p** Microspore growth and development. **q** The early mononuclear stage. **r** The late mononuclear stage. **s**, **t** Dikaryophase. **u**, **v** Matured pollen grains. **w**, **x** Anthers mature and dehiscent. Ep: epidermis, et: endothecium, ml: middle layer, ta: tapetum, mmc: microspore mother cells, dy: dyad, ca: callus, mt: microspore tetrads, m: microspore, vn: vegetative nucleus, sp.: sperm, mp: mononuclear pollen, bp: binuclear pollen, tp: trinuclear pollen. Scale bar = 20 μm in a-v; scale bar = 50 μm in w-x

In the early stage of anther development, archesporial cells with distinct nucleus and dense cytoplasm were found below the epidermis of the four corners of anther primordia (Fig. 2a). These cells further differentiated into outer primary parietal cells and inner primary sporogenous cells. The primary parietal cells continued to making periclinal and anticlinal division, enlarging the pollen sac and forming anther walls with three layers of cells (Fig. 2b). The parietal cells in the middle layer continued to divide, and finally, at the pollen mother cell (PMC) stage, the anther wall was fully differentiated and consisted of epidermis, endothecium, middle layer (one to two layers of cells), and tapetum from outside to inside (Figs. 2c). The epidermal cells were rectangular in the early stage, and gradually became elongated after the dyad and tetrad stages, and finally became irregular in the development stage of male gametophyte (Fig. 2a, o, u and x). The endothecial cells were flat rectangular at the beginning and gradually widened after the microspore stage (Fig. 2c, e, m, o, u, w and x). At the PMC stage, the middle layer changed from an initial rectangle into a thin and long strip shape, and got squeezed even more in later development (Fig. 2a, c and o-s). Tapetal cells were large, have mononuclear at first, and then developed into binucleated cells after division (Figs. 2c 2d and 2q). Tapetal cell was of the glandular type that remained in situ during the development of anther, and played an important role in microspore development (Fig. 2s and t). It gradually degenerated from the stage of mononuclear pollen grains (Figs. 2r-x). The rich materials in the middle cells and the tapetum were digested and provided nutrition for pollen during development. When the anther matured, the tapetum disappeared and the middle cells only remained (Fig. 2u, v, w and x).

### Male gametophyte development

**Fig. 3:**
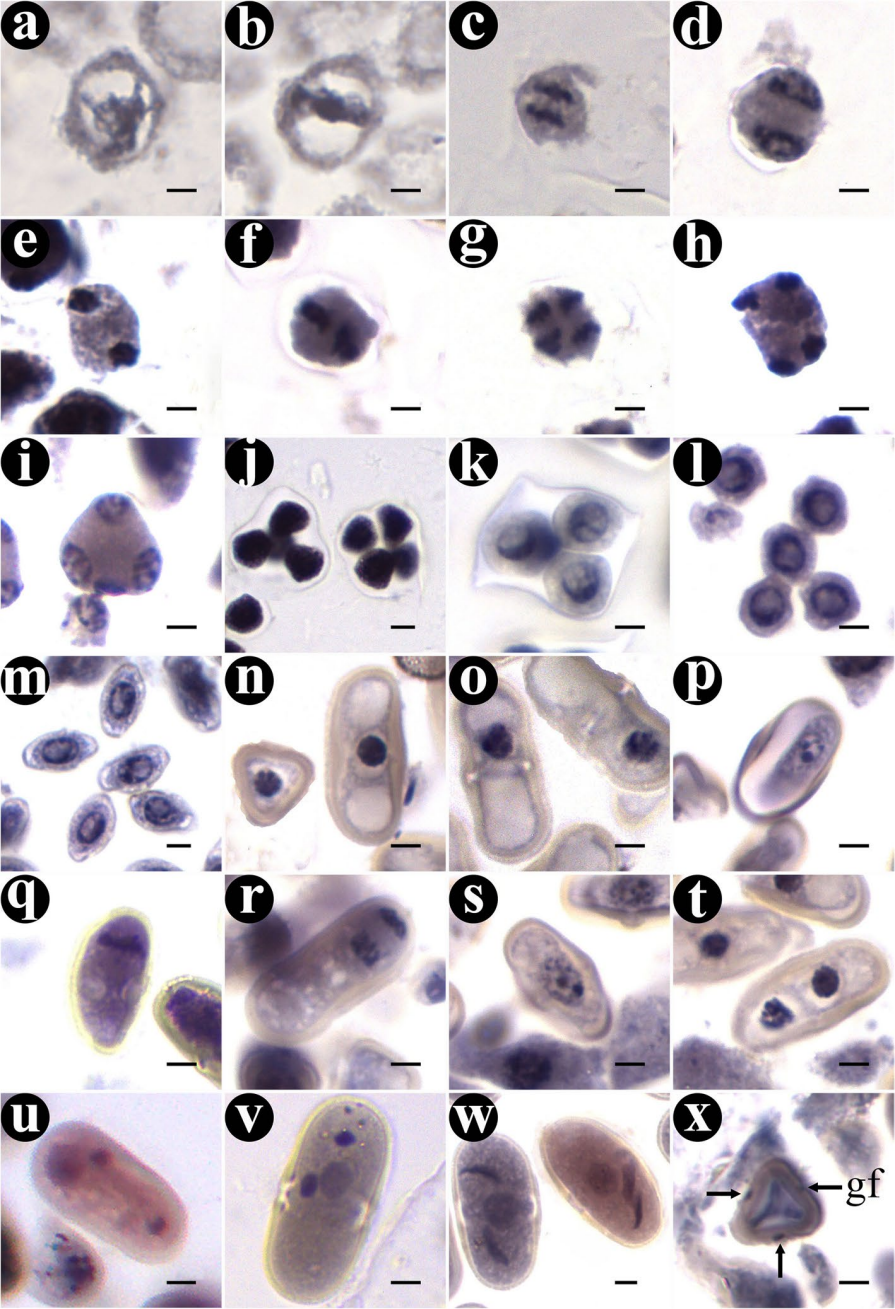
The process of microsporogenesis and male gametophyte development. **a** ProphaseI. **b** MetaphaseI. **c** AnaphaseI. **d** TelophaseI. **e** ProphaseII. **f** MetaphaseII. **g** AnaphaseII. **h** TelophaseII. **i**-**k** Tetrad of microspores. **l** Free microspores. **m** Microspores that are further growth and development. **n** Mononuclear central phase. **o** Mononuclear edge phase. **p** Prophase of mitosis. **q** Metaphase of mitosis. **r** Anaphase of mitosis. **s** Telophase of mitosis. **t** Binuclear pollen grain. **u** Germ cell division. **v**, **w** Mature pollen grain with three nucleate. **x** Three germinal furrows. Gf: germinal furrow. Scale bar = 4 μm

In the early stage of microsporogenesis, the sporogenous cells derived from archesporial cells carried out several mitotic divisions to produce a large number of microspore mother cells (MMC) which were recognizable for their large volume, dense cytoplasm and obvious nuclei (Fig. 2c). These MMC underwent two consecutive meiosis processes, including prophaseI, metaphaseI, anaphaseI, telophaseIand prophaseII, metaphaseII, anaphaseII, telophaseII (Figs. 2d-k and 3a-h). The first division resulted in dyad, and tetrad after the second division (Fig. 3d and h). There was no cell plate formed at the end of the first meiotic division, but directly entered the second meiotic division. At the end of meiosis, cytokinesis was done simultaneously and microspore tetrads were created (Fig. 3i). The majority of the tetrads was tetrahedral and surrounded by callose wall (Fig. 3j and k). Three microspores were seen on one side and another was blurred (Fig. 2l). Subsequently, the callose gradually dissolved, and the four microspores separated from each other and were released from the tetrads as free microspores (Figs. 2m, n and 3k-l).

The newly released single free microspore was round with distinct wall and dense cytoplasm. It changed to be spindle shape gradually (Figs. 3l-m). Then, with the volume increasing and cytoplasmic vacuolation occurring, the microspores entered the mononuclear central stage. At this time, the mononuclear microspore, of which the nucleus was large and located at the center of the cell, was super rectangular at the equator and triangular at the pole (Figs. 3n). As the central vacuole developed, the nucleus gradually moved to the edge of the cell (Figs. 3o). Thereafter, the microspores underwent unequal mitosis and formed two cells with different morphology and size (Figs. 3p-t). The smaller one was germ cell and the larger one was vegetative cell. The small germ cell divided further to produce two sperm cells (Fig. 3u). At this point, the mature pollen was three-celled with three germinal furrows (Figs. 3v-x).

We found that the meiosis of pollen mother cells and the development of microspore in the same flower but different anthers or in the same anther but different pollen sac were not synchronous, generally differed by one period (Fig. 2m, n, p and r).

### Ovule development

**Fig. 4:**
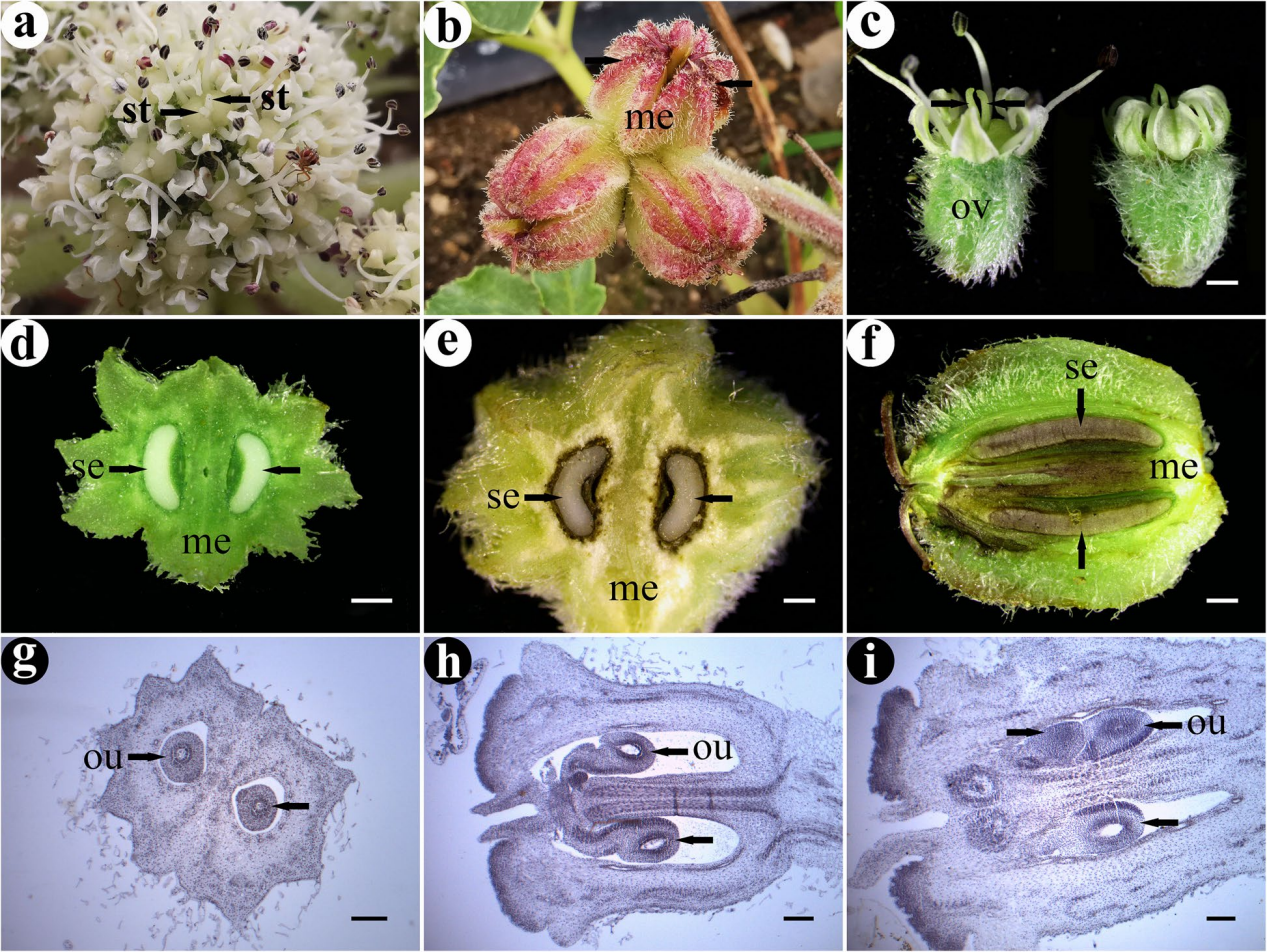
Morphology and structure of the flowers and fruits of *G. littoralis* (show pistil). **a** Flowers. **b** Fruits. **c** Flowers in stereo view. **d** Transverse section of early fruit. **e** Transverse section of late fruit. **f** Longitudinal section of fruit (The seeds and its surroundings are purple because they were stained with hematoxylin). **g** Transverse section of flower of *G. littoralis*. **h** Longitudinal section of flower of *G. littoralis*. **i** Longitudinal section of flower having tree ovules. St: style, me: mericarp, ov: ovary, se: seed, ou: ovule. Scale bar = 1 mm in c-f; scale bar = 200 μm in g-i

The flower of *G. littoralis* had two styles, and its ovary was inferior with two carpels and two ovary compartments (Fig. 4a and c-h). One ovary compartment usually had an ovule, but some had two (Fig. 4g-i). The mature ovules were anatropous, and the placenta was apical (Fig. 4h). The fruit was mericarp (Fig. 4b and d-f).

**Fig. 5:**
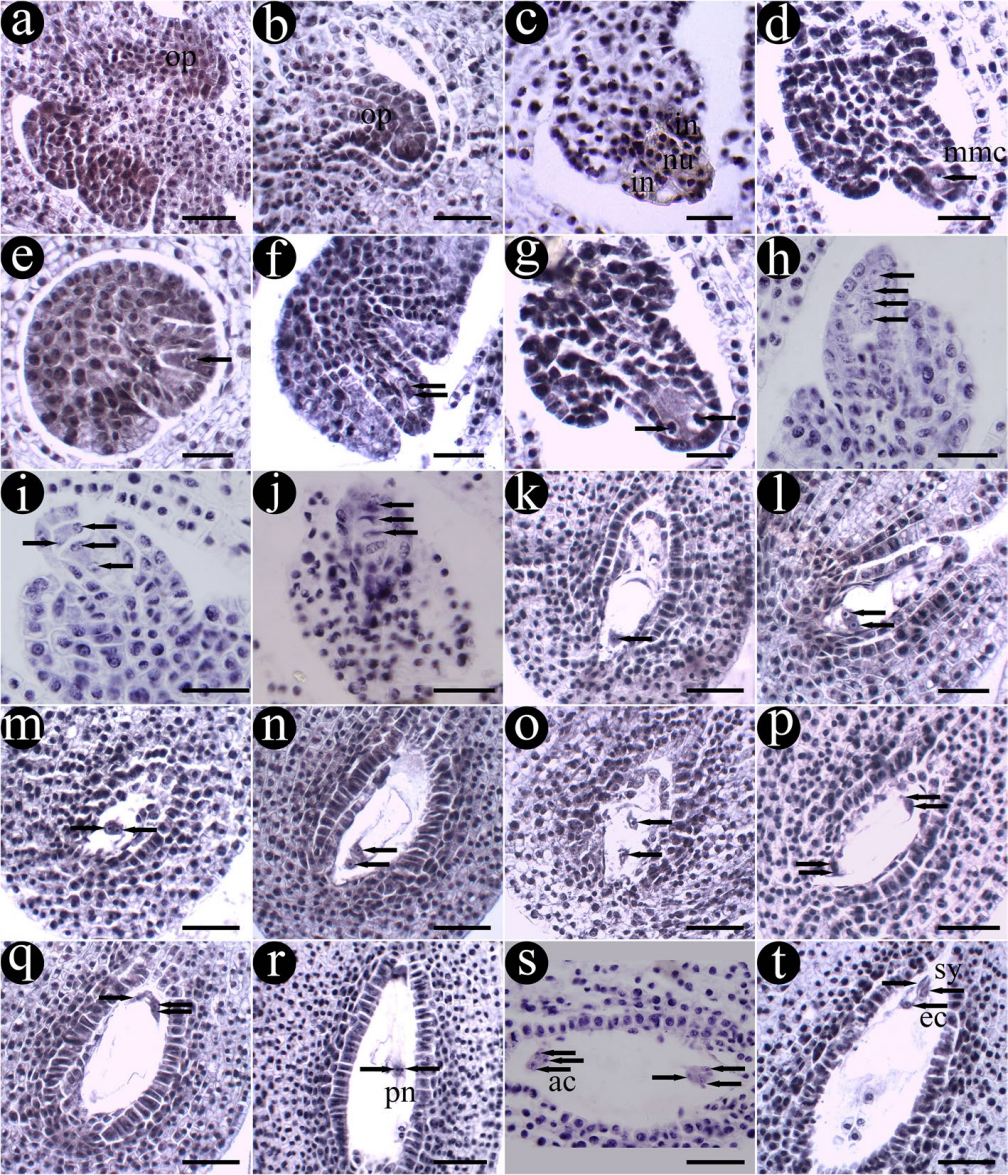
Megasporogenesis and female gametophyte development of *G. littoralis*. **a** The ovule primordial appeared. **b** Ovule primordial growth. **c** Differentiation of nucellar cells. **d** Megaspore mother cell appeared. **e** Meiotic prophase of megaspore mother cell. **f**, **g** Dyad. **h** Linear tetrad. **i** T-shaped tetrad. **j** Three megaspore at micropylar end degenerated. **k** Mononuclear embryo sac stage. **l**-**o** Binuclear embryo sac stage. **p** Tetranuclear embryo sac stage. **q**-**t** Eight-nuclear embryo sac stage. Op: ovule primordium, in: integument, nu: nucellus, mmc: megaspore mother cell, ec: egg cell, sy: synergid, pn: polar nucleus, ac: antipodal cell. Scale bar = 30 μm in c, e and g-j; scale bar = 40 μm in d, f and l; scale bar = 50 μm in a, b, k, m-r and t; scale bar = 80 μm in s

The ovule primordium was formed by a group of cells that sprout from the placenta in the ovary (Fig. 5a). The protuberance slightly curved as cells continued to divide, and differentiated into the funicle and the nucellus at the base and the upper, respectively (Fig. 5b). The epidermal cells around the base of the nucellus rapidly divided and differentiated into a single integument consisting of multiple layers of cells, while the cells inside the nucellus continued to divide and constantly increased to form a cell layer surrounding the archesporial cell (Fig. 5c). The archesporial cell was large and distinct, and had large nuclei and dense cytoplasm. After that, the embryo sac continued to develop and the development of one side of the integument accelerated, so that the ovule gradually bended and developed into an anatropous ovule finally (Fig. 4h). At this point, the integument completely enclosed the nucellus (Fig. 5k).

### Female gametophyte development

The archesporial cell with prominent nucleus and dense cytoplasm functioned as megaspore mother cell (MMC) (Fig. 5d and e). Since the MMC was surrounded by only a nucellar epidermis, the ovule of *G. littoralis* was tenuinucellate. The MMC underwent meiosis to form a dyad and then through a second division to form a tetrad which was linearly arranged or a T-shaped tetrad (Fig. 5f-i). The megaspore near the chalazal end of embryo sac developed into functional megaspore, while the other three closed to the micropylar end degenerated gradually during development (Fig. 5j).

The functional megaspore was large in size with prominent nucleus and this was mononuclear embryo sac stage (Fig. 5k). Subsequently, the mononuclear embryo sac underwent the first mitosis to form a two-nucleate embryo sac (Fig. 5l-o). The two nuclei moved toward each end of the embryo sac and then underwent a second mitosis, forming a four-nucleate embryo sac (Fig. 5p). Finally, after the third mitosis, the four-nucleate embryo sac divided into an eight-nucleate embryo sac (Fig. 5q-t). Three cells at the micropylar end constituted the egg apparatus, one of which developed into an egg cell and the other two were synergid cells. The egg cell was oval and relatively large, while the two synergids were elongated (Fig. 5q, s and t). Two cells in the middle part of the embryo sac fused with each other to form a cell with two nuclei called the polar nucleus (Fig. 5r). The three cells at the chalazal end were called antipodal cells (Fig. 5s). From then on, the embryo sac matured and consisted of seven cells and eight nuclei. Therefore, the development of the embryo sac in *G. littoralis* conformed to the *Polygonum* type.

Both female and male gametophytes underwent development for about 27 days, showing an overlap in developmental timing.

### Embryo and endosperm development of *G. littoralis*

**Fig. 6:**
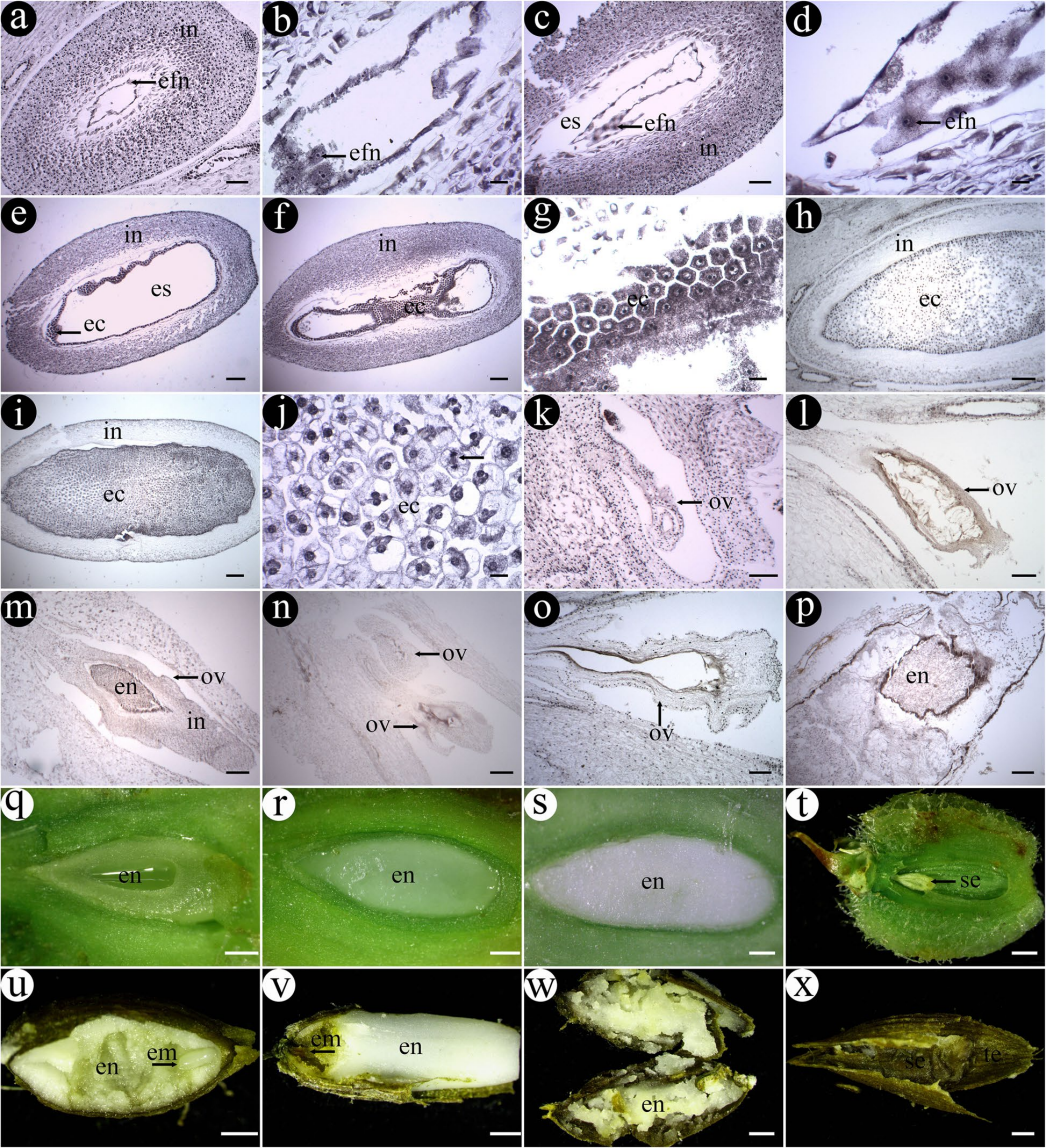
Endosperm development and abnormal seed development of *G. littoralis*. **a**, **c** The developing seed containing endosperm free nucleus. **b** Early endosperm free nuclei. **d** Late endosperm free nuclei. **e**, **f** The developing seed containing early endosperm cells. **g** Early endosperm cells. **h**, **i** Endosperm cells filled most of the seed. **j** Late endosperm cells. **k**-**p** Longitudinal section of some aborted ovules. **q**, **r**, **s** The morphology of endosperm at various developmental stages. **t** Early aborted seed. **u**, **v**, **w**, **x** Some seed abortion phenomena. En: endosperm, em: embryo, se: seed, te: testa, in: integument, es: embryo sac, efn: endosperm free nuclei, ec: endosperm cells, ov: ovule. Scale bar = 100 μm in a, c, k, l, o and p; scale bar = 20 μm in b, d, g, j; scale bar = 200 μm in e, f, h, i, m and n; scale bar = 500 μm in q-s; scale bar = 1 mm in t-x

The egg cell and the central cell in embryo sac fused with the two spermatozoa respectively to form the zygote and the primary endosperm nucleus. After that, primary endosperm nucleus carried out successive mitosis and formed endosperm free nuclei which were round, and there were variously sized nucleolus distributed freely in the cytoplasm without cell wall (Fig. 6a and b). Earlier, these free nuclei distributed around embryo sac along the integument, especially at both ends of the embryo sac. The endosperm free nuclei gradually became obvious, some of them had two or three nucleoli which could clearly observed (Fig. 6c and d). After a certain stage of mitosis, cell wall was formed among the free endosperm nuclei. From then on, the development of endosperm transitioned to the endosperm cell stage. Most of the initial endosperm cells were hexagonal and arranged neatly in embryo sac. It had dense cytoplasm and obvious nucleoli, and nucleus occupied its central position (Fig. 6e, f and g). The later endosperm cells became irregular in shape, having dark stained nucleus and smaller nucleolus. The results showed that the cells were more actively dividing and that was accompanied by cell wall formation at this time (Fig. 6h, i and j). Eventually, the inner integument of ovule gradually became thin and disappeared, and endosperm cells tightly filled most of the seed of *G. littoralis* (Fig. 6i). The artificial anatomy of fruit during this period showed that there were initially liquid to semi-liquid in the ovules and finally became solid when the endosperm fully cellulized (Fig. 6q-s).

**Fig. 7:**
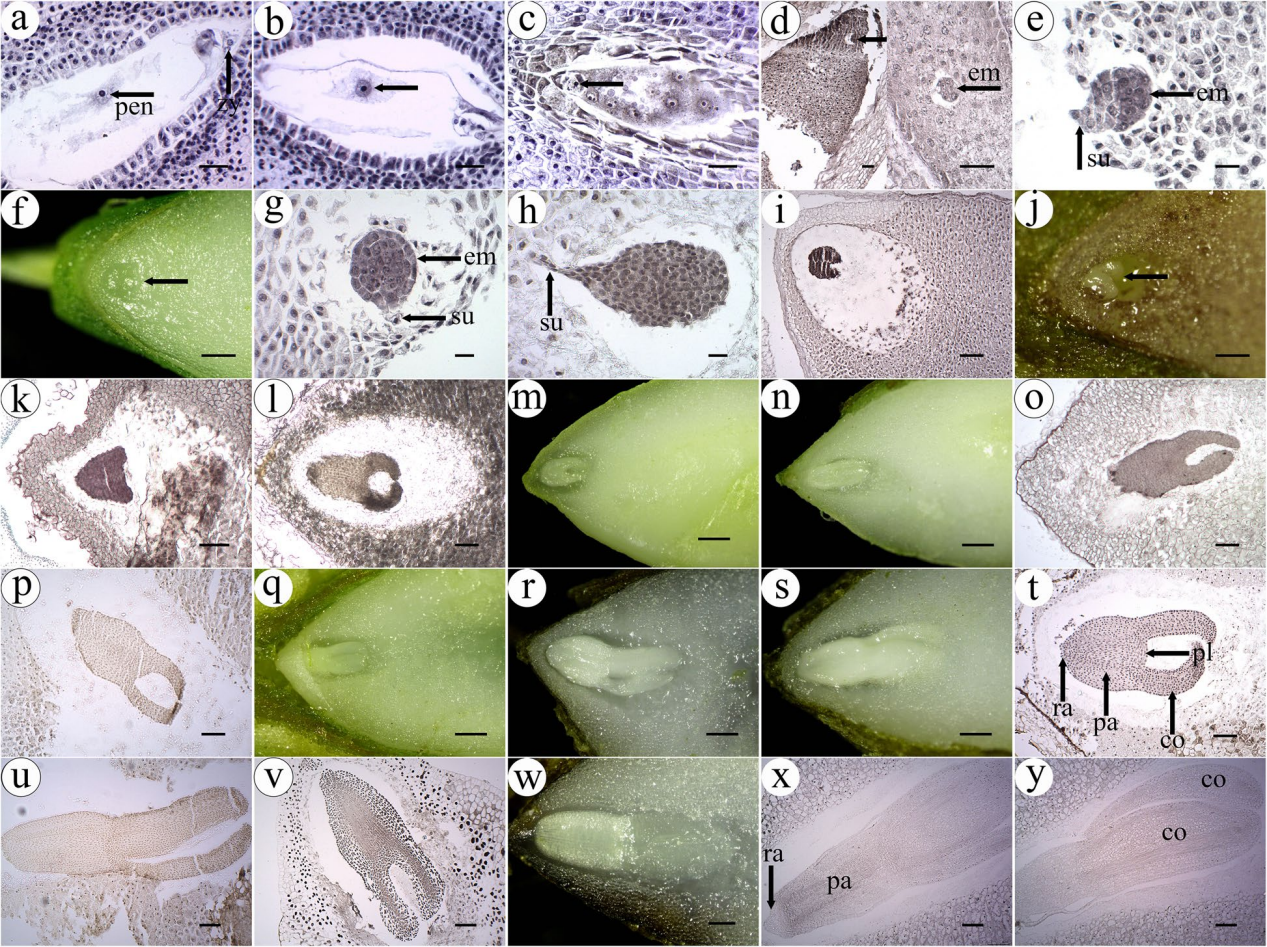
Embryo development of *G. littoralis*. **a** Fertilization. **b**, **c** Zygote. **d** Multicellular embryo. **e** Early spherical embryo. **f**, **g** Globular embryo. **h** Pear-shaped embryo. **i** Early heart-shaped embryo. **j**, **k** Heart-shaped embryo. **l** Late heart-shaped embryo. **m**, **o** Early torpedo-shaped embryo. **n**, **p** Torpedo-shaped embryo. **q**-**s**, **t**-**v** Cotyledon-shaped embryo. **w**, **x**, **y** Mature embryo. Zy: zygote, pen: primary endosperm nucleus, em: embryo, su: suspensor, ra: radicle, pa: plumular axis, pl: plantule, co: cotyledon. Scale bar = 30 μm in a-c; scale bar = 40 μm in d; scale bar = 20 μm in e, g, h; scale bar = 200 μm in f, j, w; scale bar = 100 μm in i, k-l, o-p, t-v, x-y; scale bar = 500 μm in m, n, q; scale bar = 300 μm in r, s

The zygote didn’t begin to divide before the endosperm nuclei were cellularized (Fig. 7a-c). By the time endosperm cells made up most of the developing seed, the zygote had divided into a multicellular embryo with suspensor and embryonic cells (Fig. 7d). With the further division of the embryonic cells, an early globular proembryo consisting of more than twenty cells was formed (Fig. 7e). It divided further to formed a spherical embryo, whose outer layers lined up and the inner cells more loosely clustered together (Fig. 7f and g). At this time, the endosperm cells around the embryo partially disintegrated and disappeared. The number of embryonic cells increased further and the embryo took on a pear-like shape (Fig. 7h). Then, the cell division rate on both sides of embryo increased so that it formed two protrusions, which was the early heart-shaped proembryo (Fig. 7i). Since this stage, the suspensor cells degenerated and disappeared. Endosperm cells had been disintegrated further to provide nutrients for the development of embryo, and the cavity around the embryo expanded. Then, the heart-shaped embryo formed (Fig. 7j and k-l). The protrusions on both sides of embryo elongated and a torpedo-shaped embryo formed (Figs. 7m-p). As the torpedo-shaped embryo developed further, the various parts of embryo including radicle, plumular axis, plantule and cotyledon became more distinct (Figs. 7q-v). In particular, the cells of part of the plumular axis arranged very neatly and showed an arrangement of banded layer from outside to inside. The length of the two cotyledons was approximately equal. In the mature embryo, the cotyledon was a little longer in length and thickens, and the radicle showed a cap-like structure makes it more distinct (Fig. 7w, x and y).

In addition, we also found that there were different types and degrees of abortion during the development of embryo and endosperm of *G. littoralis*. Some ovules were abnormal, with the external morphology showing atrophy of the ovules and the internal structure showing a simple structure with only a layer of integument cells and a layer of nucellar cells. In other cases, some developing seeds were abnormal, such as having small size and few endosperm cells, or abnormal embryo or endosperm development (Figs. 6k-p and t-x).

The process from zygote formation to mature embryo formation took about 48 days, much longer than the time required for the development of male and female gametophytes.

### Timeline of the development of reproductive organs in *G. littoralis*

**Fig. 8:**
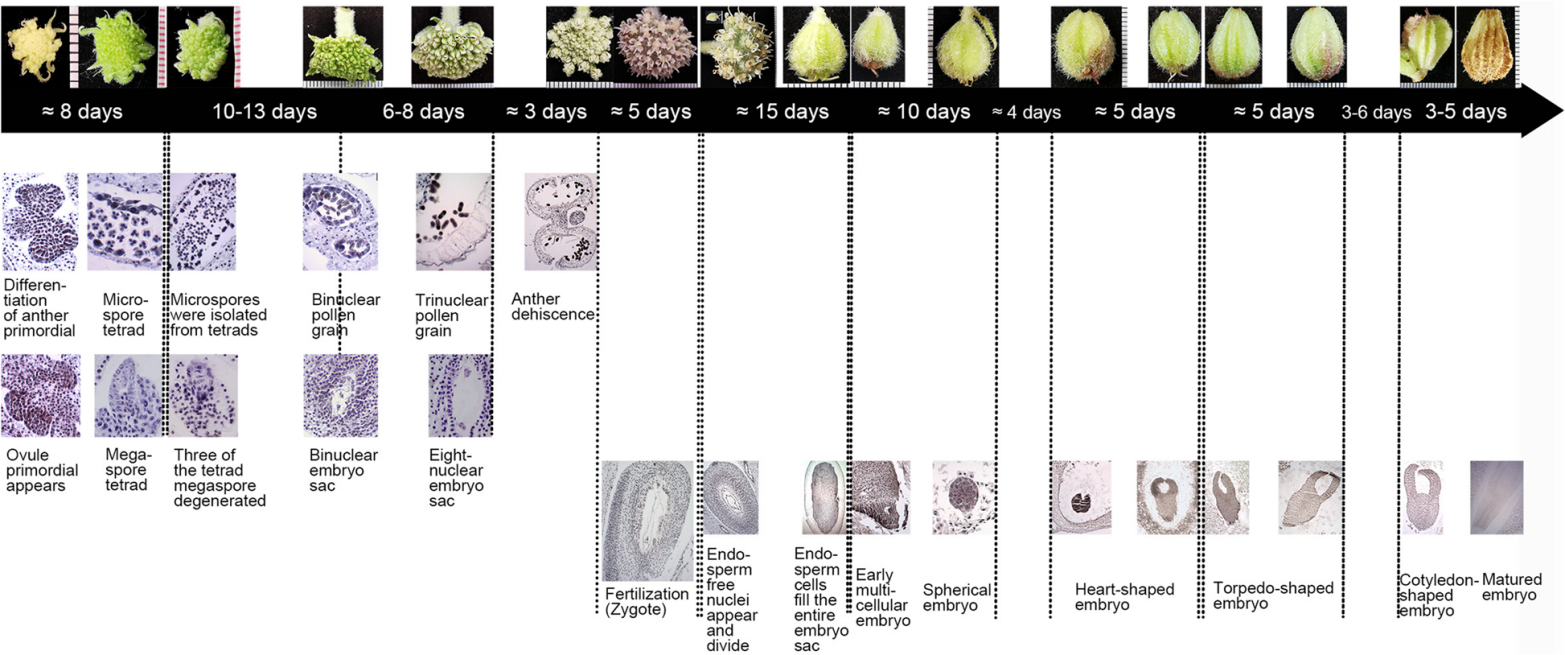
Timeline of the development of reproductive organs in *G. littoralis*. The change of petal color was accompanied by the formation of male and female gametophytes over a period of about a month. The development of embryo and endosperm was accompanied by the ripening and color change of the fruit, which took about 50 days

Sampling time was combined with the development stage of reproductive organs in *G. littoralis*, and timeline of the development was summarized (Fig. 8). The sections of male and female reproductive organs at different developmental stages were not cut from the same flower, so the time line didn’t reflect the development of pistil and stamen of the same flower, but should be viewed separately.

In early summer, occurrence of a series of events need about eight days, ranging from primitive anther differentiation to microspore tetrad formation and from the emergence of ovule primordial to the formation of megaspore tetrads. In this stage, petals color changed from the initial soft yellow to light green and stamens bent underneath the petals. Then, the microspores separated from tetrads and gradually developed into binuclear pollen. Three of the tetrad megaspores degenerated and the functional megaspore underwent a mitotic division to form two nuclei. During this stage, petals color gradually changed from light green to green, and stamens still not stretched out from petals, but the anthers can be seen a little purple at the later. This progress need about 2 weeks.

In the next 7 days, binuclear pollen grains further developed into trinucleate pollen grains and binuclear embryo sac developed into eight-nuclear embryo sac. In this stage, petals of flower lost its green color and turned white, stamens remain unstretched and anthers turned purple. Two days later, flowers gradually opened, petals color was the final white, stamens stretched out, and the anthers changed from purple to dark purple and gradually dehiscence. The two styles that initially arranged in parallel upward began to gradually separate from each other, which appeared to be ready for pollination and fertilization. However, the development of different anthers in a same flower of *G. littoralis* was not synchronized. In about 5 days, the anthers dispersed pollen, the stamens felled off, and zygote appeared over this period of time.

Formations of embryo and endosperm need about 50 days. Endosperm free nucleus divided firstly, and then endosperm cells formed and filled the embryo sac. The embryo developed later than the endosperm, early multicellular embryo, spherical embryo, heart-shaped embryo, torpedo embryo, and cotyledonous type embryo appeared sequentially, cremocarp gradually increased in size and the pericarp color changed from green to brown.

## Discussion

In this study, we explored the process of sporogenesis, gametophyte development and embryogenesis in *G. littoralis*. And the time span of some developmental stages and the corresponding external morphological characteristics and changes were summarized. In addition, we also found some abortions in flowers and seeds.

### Developmental characteristics of reproductive organogenesis

We found *G. littoralis* had dicotyledonous anther wall development, glandular tapetum, simultaneous-type microsporocyte meiosis’ cytokinesis, tetrahedral microspore tetrad, 3-celled mature pollen, tenuinucellate and anatropous ovule, polygonum-type embryo sac development. These were consistent with *Angelica sinensis*, *Angelica dahurica*, *Angelica archangelica*, *Bupleurum chinense* and *Saposhnikovia divaricate* in Apiaceae [18–22]. In addition, *G. littoralis* and most of these species had unitegmic ovules, karyotype endosperm development and the division of primary endosperm nucleus was earlier than that of zygote. However, megaspore tetrads were linear or T-shaped arrangements in *G. littoralis*, while most of megaspore tetrads reported in Apiaceae were linear. It may have phylogenetic significance that there have both primitive features such as glandular tapetum, anatropous ovule and polygonum embryo sac development and more advanced and evolutionary features like unitegmic and tenuinucellate ovules in these Apiaceae plants. Unitegmic and tenuinucellate, generally considered to be simplified types of bitegmic and crassinucellate in evolution, were reported usually accompany each other to occur in plants with sympetalae and in only part of plants with choripetalous flower [23, 24]. This could help prove that Apiaceae was a more evolved species group among plants with choripetalous flower. In addition, the apical placenta in *G. littoralis* seemed to be rare in plants with anatropous ovules, which were more often seen a basal or axial placenta [25–28]. We found that in different anthers of the same flower or different sporangium of the same anther of *G. littoralis*, the development of male gametophytes was often at different stages. Hu et al. [19] found in the study of *Angelica dahurica*, however, that meiosis of microspore mother cells in each anther chamber was in the same period, showing strict synchronization. Dyssynchrony of development of anther in *G. littoralis* may be a mechanism to prolong pollination time and promote outcrossing, which is one of its reproductive strategies [29].

### Manifestations and causes of abortion

In the life history of plants, process of reproduction and development is an important step, and problems in any part of it will affect the normal reproduction of plants and lead to abortion. Abortion can generally be classified into male, female and seed abortion.

Male sterility was mainly due to the abnormal tapeutm behavior, leading to produce some abnormal microspores or empty anthers, or affecting the anther dehiscence and subsequent fertilization process, as Guo et al. [26] found in the study of jujube. In different plant species, tapetum degeneration usually began at the tetrad stage or the later mononuclear pollen grain stage [18, 23, 30]. In the *G. littoralis* we studied, tapetum began to gradually degenerate at the stage of mononuclear pollen grain and completely degenerated and disappeared at the stage of mature pollen formation, and few abnormal conditions mentioned above were found.

Apparently, there were mainly occurred female sterility and seed abortion in *G. littoralis*. (1) Female sterility includes abnormal pistil and ovule development. Defective pistil, usually occurred in small flowers at the periphery of the inflorescence of weak plants early in flower development,  was characterized by that styles not developed to normal length or undeveloped. Abnormal developed ovules, appeared at the stage of female gametophyte development, was characterized by small and hollow embryo sac and abnormal integument. These two conditions may be explained by an imbalance in supply to organs of different parts due to nutrient limitation of the plant [31]. (2) Seed abortion occurred at different times. During pollination and fertilization stage, there were found ovaries not enlarged and have smaller ovules. It may be caused by failed pollination or fertilization. At about 6–20 days after fertilization, one of the fruits of many mericarp have been found had deformed or atrophied ovules and abnormal or degenerated endosperm. This could be ovules produced by the previous failed fertilization or caused by insufficient nutrient supply or competition during seed development. And at the late of seed development, there were found abnormal endosperm or embryo, and we speculated it may be caused by self-induced factors or unfavorable environment.

The research on abortion is still not comprehensive and profound. The study of abortion can be a direction for subsequent research, specifically analyzing the period of its occurrence, internal and external causes and patterns, and solutions, etc.

### The complete process of reproductive organ development

We linked the developmental processes to the time of acquisition of material and its state, and found that the stages of internal development events correspond to obvious external morphological changes. For example, the development of anther and ovule was accompanied by dynamic changes in petal color and stamen, the ovary began to expend after fertilization, and the developmental stages of embryo were associated with changes of pericarp. These remarkable phase characteristics will help us to infer the approximate stage of the development of flowers or fruits by looking at its external characteristics. The most important are the periods of anther dehiscence, embryo sac maturation, and pollination and fertilization. According to our research, the formation of mature embryo sac and trinucleate pollen was consistent in time, were at about May 11 to May 18 when flowers will open and turn white. Over the next few days, flowers opened and anthers turned purple or dark purple before cracking. Then, in about 5 days from May 20 to May 24, there were found the formation of zygote, indicating that this stage may be the main period of pollination and fertilization of *G. littoralis*. In breeding practice, we can whereby choose the right time of artificial pollination and propose better breeding programs to improve the efficiency and quality of breeding.

## Conclusions

Our study preliminarily revealed the embryological characteristics of reproductive organ development in *G. littoralis*. These results can serve as an important reference for studying its phylogenetic position. More importantly, these anatomical data visualized its internal development and gave us an insight into its reproduction and development, which laid a foundation for breeding work. In this study, there has not been a comprehensive list of various cases of abortion and a good explanation of the reasons for their occurrence, which needs more in-depth discussion and analysis in various aspects, so as to better solve these problems. Further study will be carried out in order to construct a comprehensive understanding of the reproductive biology of *G. littoralis*, and we hope it can be applied in the practice of cross breeding to help achieve high quality breeding after the selection of high yielding lines.

## Materials and methods

### Plant materials

*G. littoralis* seeds used in this study were provided by farmers in Laiyang, Shandong province and cultivated by us in Germplasm Resource Nursery of Yantai University. *G. littoralis* in the nursery were owned by ourselves and we had permissions to use them. The formal identification of the plants was carried out by professor Fuhua Bian from College of Life Science, Yantai University. Vouchers were stored in the life science building of Yantai University (202110BFH0168). The experimental materials were collected from 2-years or 3-years old plants in 2021 and 2022. Flowers and fruits in the different developmental stages were collected every 2 days from April to May, fixed in FAA (formalin: acetic acid glacial: 70% ethanol = 5: 5: 90, v/v) and stored at room temperature.

### Morphological observation

From mid-April, we regularly monitored the growth of *G. littoralis*. When inflorescences appeared, they were taken every 2 days, placed on an absorbent cloth with a ruler and photographed. The fruits were cut in transverse or longitudinal direction by manual slicing and observed and photographed with a stereomicroscope.

### Cytological observation of sporogenesis, gametophyte development and embryogenesis

The fixed flowers were washed twice, then stained with hematoxylin for four to 6 days (depended on the degree of development of the flower) at 45–50 °C. The fixed seeds in the different developmental stages were taken out from fruits, and soaked in sodium hydroxide solution or under running water for a period of time (adjusted according to the developmental degree of seeds), then washed twice and stained like the flowers. They were dehydrated in an ethanol gradient of 70 to 100% ethanol, transparentized in xylene, dipped in paraffin, embedded in paraffin and freeze it finally. Embedded materials were sectioned at 6-8 μm thickness using a slicer (HistoCore AUTOCUT; Leica, Germany) and the slices were treated with xylene. In the end, it was sealed with neutral balsam and the slides were observed and photographed using a light microscope equipped with camera.

## Data Availability

The data generated or analyzed in this study are included in this article.
